# Age-Diversity Practices and Retirement Preferences Among Older Workers: A Moderated Mediation Model of Work Engagement and Work Ability

**DOI:** 10.3389/fpsyg.2019.01937

**Published:** 2019-08-27

**Authors:** Inês C. Sousa, Sara Ramos, Helena Carvalho

**Affiliations:** ^1^Business Research Unit, University Institute of Lisbon (ISCTE), Lisbon, Portugal; ^2^DINÂMIA’CET-IUL, University Institute of Lisbon (ISCTE), Lisbon, Portugal; ^3^CIES-IUL, University Institute of Lisbon (ISCTE), Lisbon, Portugal

**Keywords:** retirement, older workers, age diversity, work engagement, work ability, retirement preferences, HRM, age-diversity practices

## Abstract

To meet the demographic changes, organizations are challenged to develop practices that retain older workers and encourage them to postpone retirement. The purpose of this paper is to examine the role of human resources (HR) practices in retirement preferences of older workers. Drawing on theories on lifespan development and social exchange, we suggest that organizations can facilitate longer working lives by implementing bundles of HR practices that are sensitive to age-related changes in workers’ skills, preferences, and goals – i.e., age-diversity practices. We posit that age-diversity practices are positively related to work engagement that, in turn, relates to the preference for retiring later. We further suggest that work ability moderates the relationship between age-diversity practices and work engagement. Finally, we propose a moderated mediation model in which the mediated relationship is moderated by work ability. A sample of 232 older Portuguese workers completed a questionnaire. Hypotheses were tested by using structural equation modeling (SEM). Findings show that work engagement completely mediates the relationship between age-diversity practices and the preference for early or late retirement. Moreover, this mediating relationship is more important for those workers who experience low work ability. Results further demonstrate that the effect of age-diversity practices on the preference for retiring later via work engagement is stronger for lower levels of work ability. This study highlights the organizational role in promoting longer and healthier working lives through the implementation of age-supportive HR practices.

## Introduction

People are living longer today than ever before, while fertility rates are dropping and life expectancy is increasing worldwide. In 2017, 12.7% of the world population was 60-year-old or over and this share is projected to reach 21.3% in 2050 (United Nations [UN], 2017). In Portugal, individuals aged 65 years or over will become a much larger share, rising from 21% (2016) to 35% (2070) of the population (European Commission, 2017).

As the population ages, so will the workforce. While young people tend to enter the labor force later due to longer schooling, older individuals have increased their participation in the labor market, contributing to a graying workforce (Alley and Crimmins, 2007; European Commission, 2017). However, despite that, the working-age population will shrink, as the number of younger workers entering the labor market is insufficient to replace those who are retiring (Burke and Ng, 2006; Chand and Tung, 2014). In the European Union (EU), the working-age population renewal ratio was below 100 in 2013, with 97 people aged 20 to 29 years per 100 people aged 55 to 64-year-old (Statistics Portugal, 2015). According to Statistics Portugal (2015), in 2013, Portugal was the third country in the EU-28 with the lowest working-age population renewal ratio (86 people aged 20–29 years per 100 people aged 55–64-year-old).

Demographic aging is a major challenge to which societies should respond: slower economic growth, poverty among the elderly, unsustainable pension systems, unequal distribution of saving and purchasing power, threats to the system of intergenerational reciprocity, increased costs with health care systems, and the aging and shrinkage of the labor force (Chand and Tung, 2014; Kulik et al., 2014; Nagarajan et al., 2016). In general, the aging population places intense pressure on national budgets, leading governments to implement policy changes to increase the participation rate of older workers and prevent an early exit from the workforce (Naegele and Krämer, 2002; Mahon and Millar, 2014). Some countries opted for increasing the eligible age for early and statutory retirement (e.g., Italy, Portugal, Sweden), introducing an automatic link between retirement age and life expectancy, while others opted to abolish the mandatory age for retirement for most of the occupations (e.g., Canada, United States) (Flynn, 2010; Solem et al., 2016). For example, in 10 countries of the Organization for Economic Co-operation and Development (OECD) the pensionable age is 65 years for both men and women, and in eight countries is more than 65 years (Organization for Economic Co-operation and Development [OECD], 2017).

Governments’ strategies to stimulate older people to stay in the workforce until higher age are designed and implemented at a societal level (i.e., public policies) but organizations are also called upon to participate in the promotion of fuller and longer working lives. From the organization’s perspective, the extension of working careers is crucial to cope with the expected decrease in the labor supply, ensuring their future competitiveness and sustainability (Conen et al., 2014). Labor and skills shortages require organizations to develop human resources (HR) strategies that are settled in a resource-based view: to retain the best talents and to attract and recruit valuable older workers available in the labor market (Wright et al., 2001). Therefore, organizations should assume a proactive stance to workforce aging and implement an age management strategy that encompasses analyzing the organizational HR structure (actual and desirable), including age diversity, evaluating older workers’ interests and needs, and developing age-sensitive HR practices (Walker, 1999; Vendramin et al., 2012). This approach requires the organization to be aware of workers’ abilities and preferences regarding retirement in order to implement HR practices that retain those workers who are able to perform their job and that are motivated at work and to work (Kanfer et al., 2013).

Previous investigations have examined the relationships between HR practices and retirement, showing that human resources management (HRM) can be a mechanism to prevent early retirement and to motivate older workers to work beyond retirement (e.g., Zappalà et al., 2008; Herrbach et al., 2009; Potočnik et al., 2009; Bal et al., 2012; Pak et al., 2019). However, the impact of HR practices on retirement intentions and behaviors has not been completely clarified, and the processes involved in this relationship are not yet fully understood. Thus, in this study we examine the impact of age-diversity practices on the preference for early or late retirement via work engagement, and seek to determine if this mediated relationship is moderated by work ability.

This study contributes to the literature by examining the role of age-diversity practices, a novel construct in the literature, in predicting the preference for early or late retirement. Age-diversity practices are perceived age-aware HR practices and policies that support and promote the development of workers of all ages (Walker, 1999; Boehm et al., 2014). We argue and demonstrate that such practices relate to a preference for late retirement through higher levels of work engagement. Furthermore, this study adds to the retirement literature, in particular to the field of work-related predictors of retirement, by proposing and showing work engagement as an important antecedent of the preference for late retirement. Finally, the moderated mediation model was tested through structural equation modeling (SEM), which allows researchers to better control for errors and obtain a more accurate model.

### Retirement in the Portuguese Context

The Portuguese retirement system consists of a mandatory state pension scheme administered by the Social Security organization for the private sector and the General Retirement Fund for the public sector. Individuals qualify for an old-age pension (i.e., retirement pension) based on their age and on their contributions to the social security system, which are proportional to their income. In recent years, due to the need for reducing the expenditure with the social security system, one of the major pension reforms in Portugal was linking the retirement age to life expectancy at 65-year-old (Decree-Law No. 167-E/2013). Currently, individuals must be 66 years and 5-month-old (Ordinance No. 50/2019) and have paid social security contributions for at least 15 calendar years to access the full old-age pension. In 2018 the average retirement age was 64.2-year-old, 63.4-year-old for men and 64.2 for women (PORDATA, 2019b), slightly below the statutory retirement age.

Early retirement is possible without penalties if individuals are 60-year-old and have made contributions for at least 40 years (Decree-Law No. 119/2018). Otherwise, individuals will have a penalization of 0.5% for each month of anticipation in relation to the statutory retirement age, and an additional cut of 14.67% that corresponds to the sustainability factor defined for 2019 (Ordinance No. 50/2019). Despite the penalizations, the old-age pension is still available for those workers that opt for early retirement. In 2018, 152.197 individuals were receiving anticipated old-age pensions (PORDATA, 2019a). Therefore, workers can decide to retire at the statutory retirement age, before (i.e., early retirement) or after that age.

### Theoretical Background and Research Hypotheses

Research interest in the extension of working lives has been growing in recent years. Researchers in many fields, including for example, economics and management, have focused on investigating the impacts of an aging workforce (Börsch-Supan, 2000; Kulik et al., 2014; Vasconcelos, 2015). In the HRM literature several researchers propose that organizations can take advantage of their new age structure through HR practices and policies (e.g., Kooij et al., 2008; Truxillo et al., 2015; Nagarajan et al., 2019). Previous research challenged the universality of HRM showing that work-related motives and attitudes change with age, and that there are HR practices that can especially fit older workers’ needs and preferences (Ng and Feldman, 2010; Armstrong-Stassen and Schlosser, 2011; Kooij et al., 2011; Pinto et al., 2014; Kim and Kang, 2017).

In this sense, this study proposes age-diversity practices as a strategy to retain older workers in the workforce at their best performance level and in a sustainable manner. Age-diversity practices refer to workers’ perceptions that HR practices are inclusive and non-discriminatory for individuals of all ages, as well as sensitive to age-related changes in workers’ skills, preferences and goals (Walker, 1999; Kunze et al., 2013; Boehm et al., 2014). Through the implementation of these practices, organizations communicate their purpose of promoting and maintaining an age-diverse workforce. Age-diversity practices can be understood in the light of lifespan development theories (Baltes et al., 1999). According to these theories, aging is a process of changes (e.g., physical, cognitive) that encompasses both gains and losses, and requires a remarkable individual adaptive capacity to cope with such changes (Kanfer and Ackerman, 2004; Truxillo et al., 2015). As a result of facing different changes throughout life, as people grow older, they accumulate different personal experiences, which results in more heterogeneity or differentiation within a cohort group (i.e., interindividual differences) (Kanfer and Ackerman, 2004; Carstensen, 2006). Hence, the group of older workers presents a great diversity in terms of work-related values, preferences, and interests. Age-diversity practices, understood as age-supportive HR practices, may fit different types of older workers (Truxillo et al., 2015).

Age-diversity practices focus on workers’ perceptions since there is often a discrepancy between intention and practice in HRM. In fact, previous research shows that there is a clear distinction between the organizational practices that are formulated and intended by managers and the way they are perceived by workers when implemented, leading researchers to argue that it is important to understand how workers perceive HR practices in order to examine the influence of such practices on workers’ attitudes and behaviors (Truss, 2001; Khilji and Wang, 2006). According to signaling theory, organizational practices and policies are interpreted by workers as signals of the organizations’ intentions toward them (Rynes, 1991; Casper and Harris, 2008). More specifically, HR practices emit signals of organizational interest in workers that they may interpret as perceived organizational support, facilitating the individual attachment to the organization (Den Hartog et al., 2004; Casper and Harris, 2008). Following this reasoning, HRM should create, communicate, and implement age-diversity practices to inform workers that the organization is willing to invest in the development of their potential, regardless of their age, and to maintain a long-term relationship with them.

The implementation of age-diversity practices requires the organization to assume a proactive stance in hiring, promoting, and retaining workers of all ages, and also educating managers about leading age diverse workforces (Walker, 2005; Boehm et al., 2014; Rego et al., 2017). More precisely, age-diversity practices are combinations of HR practices (i.e., HR bundles) that are age-sensitive but do not target a specific age-group, and include recruitment and selection, development and promotion, performance evaluation, work adjustment, and recognition. Furthermore, age-diversity practices are designed to create an environment where all workers, regardless of their age, can fit it and be accepted, which makes the organization attractive to future candidates. Due to their flexibility, these practices can cope with the great complexity of the different needs and goals of an age-diverse workforce, as well as remove potential age barriers.

A recent work from Kooij et al. (2014) distinguished four bundles of HR practices that potentially help to retain older workers: development, maintenance, utilization, and accommodative practices. Age-diversity practices aggregate these four bundles of HR practices, as they aim to fulfill workers’ needs by helping to improve and sustain workers’ ability, motivation, skills, attitudes, and behaviors (Kuvaas, 2008; James et al., 2011; Bal et al., 2013; Kooij et al., 2014).

Development practices refer to organizational measures that help individuals to grow and achieve higher levels of functioning, such as training and promotion (Zaleska and de Menezes, 2007; Kooij et al., 2014). Likewise, age-diversity practices are aimed at increasing older workers’ ability and motivation to work by offering opportunities to develop and apply new skills and knowledge, and also to be recognized for them. Maintenance practices are HR practices that seek to maintain older workers’ levels of functioning in the face of age-related changes (e.g., flexible work schedules) (Kooij et al., 2014). Age-diversity practices support individuals in their efforts to maintain their current levels of functioning in the face of new challenges by proposing a fair and adequate performance evaluation, which generates important feedback and thoughtful management of workers’ needs (e.g., health, skills) over time. These measures have a preventive nature and can include, for example, ergonomic adjustments or health check-ups. Utilization practices are conceptualized as those practices that, following a loss, help individuals to utilize already existing resources (e.g., experience) (Kooij et al., 2014). Similarly, when workers face a loss in their resources, age-diversity practices are aimed at recovering workers’ previous levels of functioning to ensure they will be able to perform their tasks. For instance, by valuing and recognizing workers’ experience at work (e.g., job mobility) or redesigning the job, age-diversity practices can remove highly demanding tasks that become impossible to perform and replace them with other tasks that are achievable with existing resources. Finally, accommodative practices refer to those practices that aim to meet workers’ needs by reducing their demands and help them to perform well at low levels of functioning when maintenance or recovery is no longer possible (e.g., decreases in physical workload) (Bal et al., 2013; van Dalen et al., 2015). Age-diversity practices can accommodate lower levels of functioning mainly by focusing on the adaptation of the job to workers’ needs over time, which demands attentive monitoring and follow-up by direct managers.

In a nutshell, if workers perceive the existence of age-diversity practices in their organization, they will perceive that there are HR practices that support their developing needs (i.e., development), that help them to maintain their current levels of functioning (i.e., maintenance), that make use of their existing experience, knowledge, and skills (i.e., utilization), and that help them to function well at lower levels of functioning (i.e., accommodative).

In this manuscript, we argue that the existence of such practices will be a signal to older workers that the organization is interested in retaining them. In response, workers will provide a return on the organization’s investment by showing increasing levels of work engagement that will result in a preference for late retirement. Furthermore, this relationship will be especially important for those older workers experiencing lower levels of work ability, since they feel less capable of performing their job and of remaining in the workforce for longer.

#### Age-Diversity Practices and Work Engagement

Age-diversity practices can be perceived by workers as a signal of organizational appreciation of their work. Therefore, drawing from social exchange theory (SET) (Blau, 1964; Cropanzano and Mitchell, 2005), it is possible to expect that this investment will be reciprocated by workers through increased work engagement. Work engagement has been conceptualized as a positive work-related state of fulfillment, characterized by vigor (i.e., high levels of energy while working, willingness to invest effort in work, and persistence in the presence of difficulties), dedication (i.e., strong involvement in work, and experiencing a sense of enthusiasm, inspiration, and pride), and absorption (i.e., high levels of concentration and positive engrossment in work, such that time passes quickly) (Schaufeli et al., 2002; Bakker et al., 2008).

The central premise of SET is that the exchange of resources is a fundamental form of human interaction (Cropanzano and Mitchell, 2005). Blau (1964) argues that social exchanges are voluntary actions of individuals that act in favor of another party with the expectation that such action will be reciprocated in the future. Based on this idea of reciprocal interdependence, organizational literature suggests that beneficial actions from organizations to workers contribute to the development of an expectation of some future contributions in return (Settoon et al., 1996). The implementation of organizational practices and policies, such as age-diversity practices, may create a general perception among workers that the organization values their contributions and cares for their health and well-being. If workers perceive that organizations value them and treat them fairly, they will likely reciprocate with vigor, dedication, and absorption in their work.

Age-diversity practices can foster workers’ learning and development, leading workers to reach higher levels of functioning, and therefore providing a return to the organization through increasing levels of work engagement (Bakker, 2011; Bal et al., 2013). For instance, an older worker who is included in the training about the new software will be more intrinsically motivated at work, and consequently more engaged in his/her work. Also, age-diversity practices can create adequate working conditions to help individuals meet job demands through different strategies, leading to more positive experiences at work, and therefore to an increasing self-invest in their work (Christian et al., 2011). Anticipating potential health-related changes, such as musculoskeletal disorders, the organization can, for example, redesign some physical aspects of the job by offering an adjustable table and chair, or provide training about injury prevention.

The relationship between age-diversity practices and work engagement can be understood as a social exchange between the worker and the organization that has benefits for both parties. Based on this idea, we expect that if an organization creates and implements age-diversity practices, workers will display higher levels of work engagement. In line with previous research, age-diversity practices are expected to relate to workers’ engagement. Therefore, we propose that:

Hypothesis 1: Age-diversity practices are positively related to work engagement.

#### Age-Diversity Practices, Work Engagement and the Preference for Early or Late Retirement

Age-diversity practices can be a mechanism to encourage older workers to postpone retirement. SET is a relevant theoretical rationale to explain the relationship between age-diversity practices and the preference for late retirement. A basic principle of SET is that the relationship between the organization and the worker evolves over time into a long-term commitment based on trust and loyalty, as long as the parties comply with the reciprocity rule (Cropanzano and Mitchell, 2005). Therefore, from the social exchange perspective, organizations show interest in satisfying workers’ needs and interests by implementing age-diversity practices, and workers reply reciprocally with favorable behaviors toward the organization by retiring later.

Retirement has been conceptualized as a dynamic and complex process that occurs over time and involves different factors, such as individual attributes, family aspects, job and organizational factors, or the socioeconomic context (Fisher et al., 2016; Scharn et al., 2018; Topa et al., 2018). In an attempt to understand the organizational role in the retirement decision-making process, research has identified several work-related antecedents that affect retirement, including organizational commitment (Adams and Beehr, 1998), attitudes toward work (Desmette and Gaillard, 2008), workplace timing for retirement (Feldman, 2013), and job-related stress (Wang et al., 2008). Also, the influence of HR practices in the retirement decision-making process has been extensively studied (Saba and Guerin, 2005; Herrbach et al., 2009; Potočnik et al., 2009; Lee et al., 2017). Similarly, it is expected that age-diversity practices influence retirement preferences. More specifically, we argue that if workers perceive that their organizations are developing and implementing age-diversity practices, they will likely prefer to retire later.

Despite the growing interest for research in this topic, there are still several questions to be addressed regarding the processes involved in the link between HR practices and retirement. This study proposes that work engagement is a psychological consequence of age-diversity practices and that it is a psychological antecedent of the preference for early or late retirement. In fact, research suggests that work engagement has many positive outcomes for both workers and organizations, such as job satisfaction (Saks, 2006), higher organizational commitment (Scrima et al., 2014), higher organizational performance (Christian et al., 2011), and lower intention to quit (Schaufeli and Bakker, 2004). These findings suggest that an engaged workforce can help organizations to retain their best talent (Scrima et al., 2014). Engaged workers indeed have high levels of energy, are enthusiastic about their work, and are often fully immersed in their job, so that time passes quickly. Therefore, engaged workers are intrinsically motivated to participate in the workforce, to accomplish their tasks at work, and to delay the exit from the workforce (Kanfer et al., 2013; Bentley et al., 2019). Recent evidence from de Wind et al. (2017) suggests that older workers who followed a steady low work engagement trajectory in the preceding 3 years were more likely to retire early in comparison with those who followed a steady high work engagement trajectory.

In accordance with SET, we argue that age-diversity practices, perceived by workers as an organizational investment in them, induce workers to reciprocate through increasing levels of work engagement that, in turn, will lead to a desire to retire later. Conversely, when the organization fails to offer age-diversity practices, workers are more likely to disengage themselves from the work role and leave the organization earlier through retirement. In this study, and in line with previous research, work engagement is expected to mediate the effect of age-diversity practices on the preference for early or late retirement. Therefore, we argue that:

Hypothesis 2: Work engagement mediates the relationship between age-diversity practices and the preference for early or late retirement.

#### The Moderating Role of Work Ability

Work ability has been an emerging topic in the literature on aging and work (Morelock et al., 2017; Cadiz et al., 2018). Although there are different conceptualizations of work ability, in this study it is defined as an individual’s self-assessment about the degree to which he or she has personal resources to meet the demands of work (McGonagle et al., 2015). Individuals experiencing high levels of work ability perceive themselves as having the functional capacities (mental, physical, and social resources) and individual health, competence, attitudes, and values to successfully manage and perform the work tasks (Tengland, 2011; Ilmarinen and von Bonsdorff, 2016). On the contrary, workers who perceive that their resources are not adequate to meet the job requirements are experiencing low work ability.

Earlier research demonstrates that work ability is influenced by individual (e.g., gender, personality) and work-related (e.g., organizational practices, physical and mental demands) factors (Tuomi et al., 2004; McGonagle et al., 2015). Therefore, organizations can play an important role in promoting the work ability of their workers. In line with selection, optimization, and compensation (SOC) theory (Baltes and Baltes, 1990), organizations can implement practices that help older workers to cope with age-related losses that will probably affect their ability to meet job requirements. Such practices can help to reduce the gap between individuals’ abilities and job demands by allowing the use of SOC strategies that maximize gains and minimize losses. For example, coworker and supervisor support (Mazloumi et al., 2012), decreased work demands (Tuomi et al., 2004), and effort-reward balance (Fischer and Martinez, 2012) can contribute to better work ability among older workers. In addition to the extensive body of research examining work ability as an outcome, there are also several studies investigating work ability as a predictor. Tuomi et al. (2001) suggest that workers experiencing good work ability are more likely to produce high quality work, to achieve higher levels of productivity, and to enjoy staying in the job. Therefore, good work ability is associated with positive work-related outcomes that are beneficial for the organization.

Previous research also shows that work ability is positively associated with work engagement (Airila et al., 2012; Rongen et al., 2014). Although work engagement is usually identified as an antecedent of work ability, some researchers acknowledge that this relationship may be reciprocal (Cadiz et al., 2018). Engagement encompasses high levels of vigor, dedication, and absorption, and it is likely that work ability influences the extent to which people are able to be involved in their work while demonstrating high levels of energy, enthusiasm, and concentration. Thus, different levels of work ability can moderate the positive impact of age-diversity practices on work engagement.

Based on these arguments, it is expected that older workers who perceive low work ability will attribute more importance to the role of age-diversity practices in retirement. Because older workers with low work ability perceive that they possess less functional capacity to meet job demands, they are more likely to perceive that age-diversity practices will facilitate their engagement. On the contrary, workers experiencing high work ability will feel more capable of performing their tasks and perceive age-diversity practices as less important for them to be fully engaged in work. Accordingly, this paper proposes that the relationship between age-diversity practices and work engagement is moderated by work ability. Therefore, we argue that:

Hypothesis 3: Work ability moderates the positive relationship between age-diversity practices and work engagement, such that the relationship is stronger among workers perceiving low work ability.

Following the previous reasoning, this study also hypothesizes that work ability moderates the strength of the mediated relationship between age-diversity practices and the preference for early or late retirement through work engagement. When older workers experience low work ability, they interpret organizational practices in a positive way and consequently may reciprocate by being fully engaged in their work (Alfes et al., 2013), which leads to a preference for retiring later. The perception of incapacity associated with low work ability reinforces the need to implement age-diversity practices that contribute to an engaged workforce and motivate the preference for a later retirement age. On the other hand, older workers who experience high levels of work ability already feel that they are able to meet job demands, and thus are more engaged in work and more willing to retire later. Therefore, for workers with high work ability, the indirect effect of age-diversity practices on the preference for early or late retirement is weaker. The following hypothesis is then proposed:

Hypothesis 4: Work ability moderates the indirect effect of age-diversity practices on the preference for early or late retirement via work engagement, such that the positive indirect effect is stronger among workers perceiving low work ability.

**FIGURE 1:**
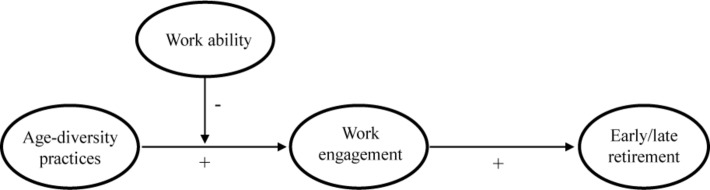
Proposed theoretical model.

In summary, this study examines the impact of age-diversity practices on work engagement that, in turn, will influence the preference for early or late retirement. Also, this investigation argues that the relationship between age-diversity practices, work engagement, and the preference for early or late retirement could be strengthened by a negative perception of work ability. Figure 1 presents the theoretical model of this study.

## Materials and Methods

### Procedure and Sample

The data used here were obtained as part of a larger survey about work attitudes and retirement among Portuguese workers. The questionnaire had both an online and a paper and pencil version, and participants took on average 15 min to complete it. Ten large companies from different industries (e.g., energy, retail, manufacturing, services) and both public and private sectors were invited to disseminate the study and ask for voluntary participation among their workers. Data were also collected by a group of undergraduate and master students from ISCTE – University Institute of Lisbon, through their personal contacts and in their workplaces and internship places. Students participated in this research as part of their academic training and earned credit for completing the evaluation.

A sample of 232 older Portuguese workers, aged between 50 and 72-year-old (*M* = 55.08, *SD* = 4.52), completed the questionnaire. Among the participants, 56.5% were female and 47.4% had completed higher education. More than half of the participants (64.2%) were the primary wage earner in their households. Twenty-two percent of them worked in the Education and training sector, 14.7% in the Health and social support sector, and 13.4% in Manufacturing and production. The majority of participants (51.7%) worked in the public sector. Regarding organizational tenure, 17.7% of the participants were in the organization for 10 years or fewer and 28.9% for more than 30 years. Ninety-two percent of the sample had worked for more than 25 years.

### Measures

All participants were fluent in Portuguese, which required the translation of the items from the source language (i.e., English) to the target language (Portuguese). We first translated the questionnaire to Portuguese, and then an independent researcher performed a blind back-translation to English (Brislin, 1970; van de Vijver and Hambleton, 1996). The back-translation version was compared to the original version and evaluated by a bilingual researcher, who did not suggest any modifications. Given that the work engagement scale was already adapted and validated for Portugal (Sinval et al., 2018), it was not included in the translation process.

Age-diversity practices were measured using a seven-item scale that assesses the extent to which workers perceive an inclusive and non-discriminatory treatment of workers of all ages regarding age-sensitive HR practices. Four of these items were retrieved from Boehm et al. (2014) age-diversity climate scale (2014). We included three additional items to account for organizational practices related to (a) performance evaluation (“All workers have the same opportunities to get an adequate evaluation, regardless of their age”), (b) recognition (“Experience, skills, and knowledge of workers are recognized, irrespective of their age”), and (c) job design (“The work is adjusted to workers’ needs over time”). Responses were rated on a six-point scale ranging from 1 (strongly disagree) to 6 (strongly agree). Additionally, a Confirmatory Factor Analysis (CFA) was conducted to test the one-factor structure of this scale. The results revealed a satisfactory model fit: χ^2^ = 26.08, *df* = 9; χ^2/^*df* = 2.898; CFI = 0.98; TLI = 0.95; RMSEA = 0.09; SRMR = 0.03 (Hu and Bentler, 1999). The Cronbach’s alpha showed a good internal reliability (α = 0.87).

Work ability was assessed through four questions regarding both physical and mental demands of the work, at present and in the future (adapted from Tuomi et al., 1998; Weigl et al., 2013; McGonagle et al., 2015). It refers to an individual, subjective perception about workers’ own ability to continue working considering work demands and personal resources (McGonagle et al., 2015). A sample item is: “How do you rate your current work ability to meet physical demands?” Participants answered on a five-point scale ranging from 1 (very poor) to 5 (very good). The internal consistency coefficient for this scale was good (α = 0.90).

Work engagement was measured using the short version of the Utrecht Work Engagement Scale with nine items (UWES-9) (Schaufeli et al., 2006; Sinval et al., 2018). The scale assesses a positive, fulfilling work-related state of mind (Schaufeli et al., 2002). An example item is: “I am enthusiastic about my job.” Participants indicated how often they felt the way described in the statements on a seven-point rating scale ranging from 0 (never) to 6 (always). The scale revealed a good internal consistency (α = 0.95).

Preference for early or late retirement was obtained through two questions. Participants indicated their expected (“At what age do you expect you could retire?”) and preferred (“At what age would you like to retire?”) retirement age (Zappalà et al., 2008; Hess, 2018). Preferred and expected retirement age were positively correlated (*r* = 0.50, *p* < 0.001), without multicollinearity problems as proposed by Tabachnick and Fidell (2013). To obtain the preference for early or late retirement, we calculated the difference between these two items by subtracting the expected retirement age from the preferred retirement age. As suggested by Hess (2018) and Zappalà et al. (2008), a negative value indicated a preference for early retirement (i.e., workers perceive they will have to work longer than they would like to), while a positive value revealed a preference for late retirement (i.e., workers would like to work longer than the statutory retirement age but there are factors preventing this desire).

### Control Variables

Age, gender, education, status as wage earner, and organizational tenure were assessed as control variables. Age has been identified as a significant predictor of work ability (van den Berg et al., 2009; Converso et al., 2018), work engagement (Kim and Kang, 2017), and retirement planning (Evans et al., 1985; Taylor and Shore, 1995). Earlier research shows that gender, education, income, and organizational tenure are related with the transition to retirement (Wang and Shultz, 2010; Fisher et al., 2016; Topa et al., 2018). Age was measured in years. Gender and education were coded as dummy variables (0 = male, 1 = female; 0 = without an academic degree, 1 = with an academic degree, respectively). Participants were asked if they were the primary wage earner (i.e., if their job was the main source of income for the household) (0 = no; 1 = yes). Finally, organizational tenure was also dummy coded (0 = ≤ 30 years; 1 = > 30 years).

### Data Analysis

A CFA was conducted to validate the measurement model. To test the moderated mediation, SEM was used. Bootstrapping was also implemented to validate the results obtained by the parametric method (maximum likelihood estimation). We used 5,000 bootstrap samples to generate 95% bias-corrected confidence intervals (CI) for both direct and indirect effects (Cheung and Lau, 2008).

Considering the model’s complexity, due to the number of indicators involved in the moderation and that can result in an under identified model (Cortina et al., 2001), we followed the matched-pair strategy for defining products to represent the latent interaction, as suggested by Marsh et al. (2004). We began the analysis by standardizing all the indicators of age-diversity practices (independent variable) and work ability (moderator). Then we created the multiplicative terms of the latent variable interaction factor matching the items in terms of their quality. As age-diversity practices have seven items and work ability has four, we created four pairs, matching the best four indicators from age-diversity practices with all the indicators from work ability (i.e., the best indicator from age-diversity practices with the best indicator from work ability, etc.) (Marsh et al., 2004). Finally, we tested the research hypotheses under study. The analyses were performed with Analysis of Moment Structures (AMOS, v. 24; Arbuckle, 2016).

### Measurement Model and Common Method Variance

The measurement model specifies the relationships between latent variables and their indicators (Henseler, 2017). Table 1 shows the model fit statistics for different measurement models. The baseline three-factor model showed an adequate model-data fit. The normed chi-square was 2.08 (χ^2^ = 317.85, *df* = 156; χ^2/^*df* = 2.083), below the cutoff value of 3 (Hair et al., 2010). The comparative fit index (CFI) and Tucker-Lewis index (TLI) values were 0.96 and 0.95, respectively, near the suggested cutoff value of 0.95 (Hair et al., 2010). The root-mean square error of approximation (RMSEA) was 0.07, with the 90% confidence interval for RMSEA ranging from 0.056 to 0.078, which was less than the 0.08 value suggested by Browne and Cudeck (1992), and indicates a reasonable fit. The standardized root-mean square residual (SRMR) was 0.05, a value smaller than 0.08, and that indicates a good fit (Hu and Bentler, 1999; Hair et al., 2014).

**TABLE 1:** Fit indices for measurement model comparison.

**Models**	**Three-Factor – Model 1 (Full measurement model)**	**Model 2^a^**	**Model 3^b^**	**Model 4^c^**	**Model 5^d^ (Harman’s single factor)**
χ^2^ (df)	317.85 (156)	459.18 (155)	422.73 (154)	469.88 (151)	482.74 (149)
χ^2^/df	2.083	2.962	2.745	3.112	3.240
CFI	0.96	0.89	0.93	0.92	0.92
TLI	0.95	0.91	0.92	0.90	0.89
RMSEA	0.07	0.09	0.09	0.10	0.10
SRMR	0.05	0.06	0.12	0.09	0.09
χ^2^_dif_ (df)		141.33 (1)^∗∗∗^	104.88 (2)^∗∗∗^	152.03 (5)^∗∗∗^	164.89 (7)^∗∗∗^

**N* = 232; χ^2^, chi-square; df, degrees of freedom; χ^2^/df, normed chi-square; CFI, comparative fit index; TLI, Tucker–Lewis index; RMSEA, root mean square error of approximation; SRMR, standardized root mean square residual; χ^2^_*dif*_, chi-square difference. ^∗∗∗^*p* < 0.001. ^*a*^Work ability and Work engagement combined into a single factor. ^*b*^Work ability and Age-diversity practices combined into a single factor. ^*c*^Work engagement and Age-diversity practices combined into a single factor. ^*d*^The three factors combined into a single factor.*

As shown in Table 1, results demonstrated that the hypothesized measurement model (three-factor model) shows a better fit than all the alternative models. All the standardized loadings are greater than 0.50 (as suggested by Hair et al., 2010), and range from 0.63 to 0.94. Therefore, we proceeded with the assessment of the reliability and construct validity (convergent and discriminant validity) of the hypothesized measurement model. Convergent validity results are summarized in Table 2.

**TABLE 2:** Measurement model: convergent validity.

	**CR**	**AVE**	**MSV**
Age-diversity practices	0.88	0.51	0.30
Work ability	0.90	0.69	0.32
Work engagement	0.95	0.70	0.32

*CR, composite reliability; AVE, average variance extracted; MSV, maximum shared variance.*

Cronbach’s alpha, composite reliability (CR), and average variance extracted (AVE) were used for evaluating convergent validity. As presented earlier, Cronbach’s alphas, which ranged from 0.87 to 0.95, showed that the latent variables had very good/excellent reliability (Kline, 2011). As shown in Table 2, the CR values ranged from 0.88 to 0.95, exceeding the minimum reliability value of 0.70, and all of the AVE values were above the threshold value of 0.50, confirming the construct reliability of composite indicators (Hair et al., 2010). The latent variables therefore meet the standard requirement of convergent validity. The assessment of discriminant validity uses the Fornell-Larcker criterion, which defines that the square root of each construct’s AVE should be greater than the inter-construct correlations (Fornell and Larcker, 1981). The Fornell-Larcker criterion is met with regard to all measures. Also, discriminant validity is obtained if the AVE is greater than the maximum shared variance (MSV) (Hair et al., 2010), which is shown in Table 2. These results sustain the existence of discriminant validity in the model.

In this study, all variables were collected from a single source at one point in time and using the same self-report questionnaire. Thus, we needed to establish whether common method bias was a concern in our data (Williams and Brown, 1994; Podsakoff et al., 2003). As we examined individual perceptions (about age-diversity practices and work ability), dispositions (work engagement), and preferences (for early or late retirement), participants are the best source of data regarding their own beliefs. Other sources (e.g., supervisors, coworkers) would have difficulty providing responses on behalf of workers due to the subjective nature of the variables, making self-reported measures clearly more appropriate (Conway and Lance, 2010). Nevertheless, we conducted Harman’s single-factor test using CFA (Podsakoff et al., 2003). Results showed that a single-factor model did not fit the data well (Table 1). These results show that common method variance is not a major concern in this study.

## Results

Table 3 presents descriptive statistics (mean and standard deviation), reliability and bivariate correlations for all of the constructs. Participants revealed a preference for retiring on average at 61.06-year-old (*SD* = 4.77), an average of 4.16 years before their expected retirement age (*M* = 65.22, *SD* = 3.72). Age and organizational tenure were significantly correlated with the preference for early or late retirement (*r* = 0.35, *p* < 0.001; *r* = 0.18, *p* < 0.01, respectively). Gender, education, and status as wage earner showed a significant correlation with work ability (*r* = −0.19, *p* < 0.01; *r* = 0.17, *p* < 0.01; *r* = 0.13, *p* < 0.05, respectively). A significant correlation was found between status as wage earner and work engagement (*r* = 0.14, *p* < 0.05). Age-diversity practices, work ability, and work engagement had a significant correlation with preference for early or late retirement (*r* = 0.19, *p* < 0.01; *r* = 0.17, *p* < 0.01; *r* = 0.24, *p* < 0.001, respectively).

**TABLE 3:** Construct means, standard deviations, correlations, and reliabilities.

	***M***	***SD***	**1**	**2**	**3**	**4**	**5**	**6**	**7**	**8**
(1) Age	55.08	4.52								
(2) Gender^a^	0.56	0.50	–0.06							
(3) Education^b^	0.47	0.50	0.10	0.05						
(4) Primary wage earner^c^	0.64	0.48	0.05	–0.27^∗∗∗^	0.04					
(5) Organizational tenure^d^	0.29	0.46	0.34^∗∗∗^	–0.01	–0.02	0.08				
(6) Age-diversity practices	3.64	1.18	0.04	–0.07	0.13	0.11	0.02	(0.88)		
(7) Work ability	3.69	0.82	–0.04	–0.19^∗∗^	0.17^∗∗^	0.13^∗^	–0.05	0.35^∗∗∗^	(0.90)	
(8) Work engagement	4.57	1.36	0.09	–0.06	0.08	0.14^∗^	0.04	0.50^∗∗∗^	0.60^∗∗∗^	(0.95)
(9) Early/late retirement	–4.16	4.36	0.35^∗∗∗^	–0.11	0.03	0.10	0.18^∗∗^	0.19^∗∗^	0.17^∗∗^	0.24^∗∗∗^

**N* = 232. Reliability coefficients are reported in parentheses. ^*a*^Male = 0, Female = 1. ^*b*^Without an academic degree = 0, with an academic degree = 1. ^*c*^ No = 0, Yes = 1. ^*d*^ ≤30 years = 0, >30 years = 1. ^∗^*p* < 0.05; ^∗∗^*p* < 0.01; ^∗∗∗^*p* < 0.001 (two-tailed test).*

### The Structural Model

The structural model specifies the relationships between the constructs (Henseler, 2017). Our hypothesized moderated mediation model had an acceptable fit to the data: χ^2^ = 523.78; *df* = 261; χ^2^/*df* = 2.007; CFI = 0.94; TLI = 0.93; RMSEA = 0.07; SRMR = 0.09. Age, gender, education, status as wage earner, and organizational tenure were controlled but they did not change the estimated main effects and interaction effects in the moderated mediation model.

Hypothesis 1 posited that age-diversity practices would be positively related to work engagement (Table 4). Results support this hypothesis (*B* = 0.71, *p* < 0.001, 95% CI = 0.48; 0.97), showing that as age-diversity practices increase, work engagement also increases. Hypothesis 2 stated that work engagement would mediate the relationship between age-diversity practices and the preference for early or late retirement. Results revealed the indirect effect to be significant (*B* = 0.51, 95% CI = 0.13; 1.04), providing support for Hypothesis 2. The direct effect became not significant (*B* = 0.47, *p* > 0.05, 95% CI = −0.67; 1.56), suggesting a full mediation (Preacher and Hayes, 2004).

**TABLE 4:** Multiple Regression Results for Work engagement and Preference for early or late retirement.

**Variables**	**Work engagement**			**Preference for early or late retirement**		
	**Coefficient**	**SE**	**95% CI**	**Coefficient**	**SE**	**95% CI**
**Independent variables**						
Age-diversity practices	0.71^∗∗∗^	0.13	0.48, 0.97	0.98^∗^	0.48	0.03, 1.88
Work ability	0.79^∗∗∗^	0.12	0.56, 1.06			
**Interaction**						
Age-diversity practices × Work ability	−0.28^∗^	0.12	−0.55, −0.03			
**Mediator**						
Work engagement				0.71^∗^	0.29	0.15, 1.28
**Direct effect**						
Age-diversity practices				0.47	0.57	−0.67, 1.56
**Indirect effect**						
Age-diversity practices				0.51^∗∗^	0.23	0.13, 1.04

**N* = 232. All estimates for the moderated mediation were also tested for significance using bias-corrected (BC) confidence interval from 5,000 bootstrap samples. ^∗^*p* < 0.05; ^∗∗^p < 0.01; ^∗∗∗^*p* < 0.001.*

**FIGURE 2:**
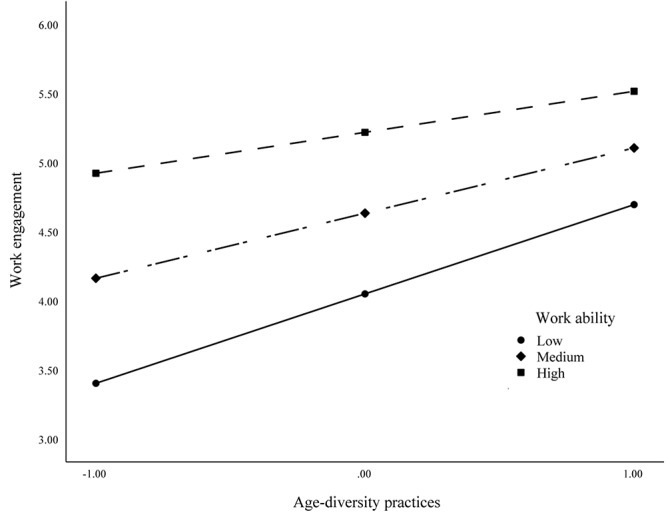
Moderating effect of work ability on the relationship between age-diversity practices and work ability.

Hypothesis 3 specified that work ability would moderate the relationship between age-diversity practices and work engagement. As shown in Table 4, the interaction term of age-diversity practices and work ability was significantly associated with work engagement (*B* = −0.28, *p* = 0.03, 95% CI = −0.55; −0.03). To further examine the moderation effect, we plotted the results and performed a simple slopes analysis based on the mean of the moderator (work ability) and at one SD above and below the moderator’s mean (Aiken and West, 1991; Preacher et al., 2006). Figure 2 plots the interaction, which shows that as work ability decreases, the effect of age-diversity practices on work engagement increases. The simple slopes test further supports the moderation effect. The relationship between age-diversity practices and work engagement is stronger when work ability is low (*B* = 1.00, 95% CI = 0.64; 1.36) and weaker when work ability is high (*B* = 0.43, 95% CI = 0.08; 0.78), supporting the third hypothesis.

**FIGURE 3:**
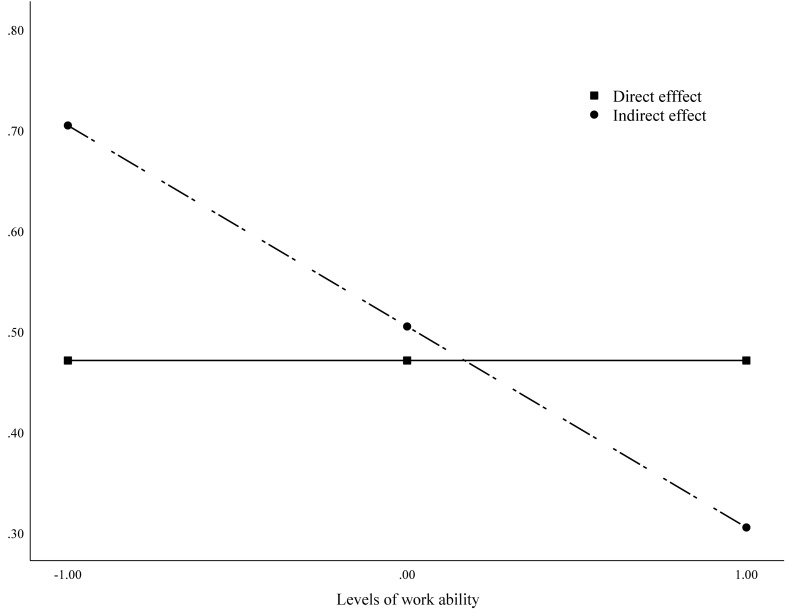
The conditional indirect effect of age-diversity practices on the preference for early or late retirement via work engagement.

Hypothesis 4 posited that work ability would moderate the indirect effect of age-diversity practices on the preference for early or late retirement via work engagement. Findings showed that the index of moderated mediation was −0.20 (95% CI = −0.56; −0.02), demonstrating that as work ability decreases, the indirect effect of age-diversity practices on the preference for early or late retirement via work engagement increases (Figure 3). The simple slopes test provides further support for Hypothesis 4 (B_low_ = 0.70, 95% CI = 0.18; 1.46; B_high_ = 0.31, 95% CI = 0.06; 0.83).

**FIGURE 4:**
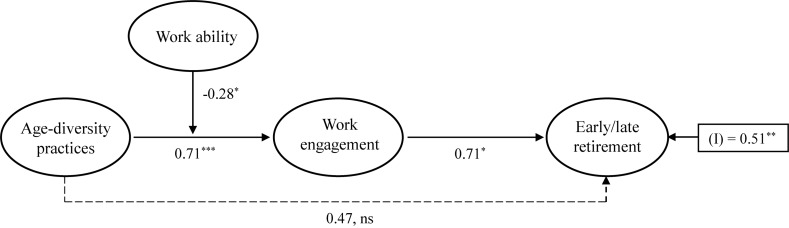
Results of the moderated mediation model. (I) Indirect effect of age-diversity practices on the preference for early or late retirement via work engagement; ns: not significant; ^∗^*p* < 0.05; ^∗∗^*p* < 0.01; ^∗∗∗^*p* < 0.001.

Overall, work engagement mediates the relationship between age-diversity practices and the preference for early or late retirement, and this indirect effect was especially important for older workers who experience low work ability (Figure 4).

## Discussion

In response to the calls for identifying the factors that facilitate the extension of working lives (Burke and Ng, 2006; Vendramin et al., 2012), this study explored processes of mediation and moderation linking age-diversity practices to the preference for early or late retirement, in a sample of older workers. We proposed that age-diversity practices influence work engagement that, in turn, will influence the preference for early or late retirement, and, further, that the strength of this relationship is moderated by workers’ perceptions of work ability. Data supported our moderated mediation model.

The first hypothesis proposed that age-diversity practices would be positively related to work engagement, and it was supported by the findings. Older workers that perceived the existence of age-diversity practices in their organization felt higher levels of energy, were more dedicated to their work, and were more often fully immersed in work. As stated by the reciprocity rule of SET, workers perceive age-diversity practices as a signal of organizational intention for a long-term investment in them and reciprocate by increasing their involvement in their work. This finding is consistent with those of previous studies showing that HR practices influence workers’ involvement in work (Alfes et al., 2013; Conway et al., 2016). In our study, we narrow the broad range of HR practices by introducing age-diversity practices as an antecedent of work engagement. Older workers are a heterogeneous group and differ in their particular needs and expectations regarding their work. The implementation of HR practices that are age-sensitive (i.e., sensitive to the diversity that results from individual experiences throughout life) can promote older workers’ engagement by responding to these specific needs and expectations. For example, workers who seek growth and want to achieve higher levels of functioning will feel more engaged in their work if they receive regular training or perceive the existence of opportunities for internal mobility.

The second hypothesis posited that work engagement would mediate the positive relationship between age-diversity practices and the preference for early or late retirement. This hypothesis was also sustained by the results. Individuals who have a positive perception of age-diversity practices are more likely to be engaged in their work and, therefore, have a greater tendency to remain in the workforce, delaying retirement. This result shows a mutual investment in the employee-organization relationship that engenders feelings of obligation, gratitude, and trust in both parties (Blau, 1964). This sense of commitment can buffer the “proximity to retirement” effect (Ekerdt and DeViney, 1993) that is characterized by a period of a decreasing investment in work-related activities (de Wind et al., 2017). Therefore, organizational factors such as age-diversity practices can slow down the preretirement work disengagement process by creating the perception that older workers’ talent is valued and needed in the organization (Damman et al., 2013). Hence, age-diversity practices might increase the likelihood that older workers will extend their working careers and retire later.

In the third hypothesis, we proposed that work ability would moderate the relationship between age-diversity practices and work engagement, and this, too, was supported by the empirical findings. The extent to which age-diversity practices lead to work engagement is contingent upon the overall levels of functioning of older workers. More specifically, results demonstrate that the influence of age-diversity practices on work engagement can be especially important for older workers feeling less capable to meet job requirements. As work ability is related with age-related changes that occur throughout life, workers will likely experience decreasing levels of work ability as they age (Converso et al., 2018), which makes age-diversity practices especially important for older workers’ engagement. As the aging process is different from person to person, due to the multiplicity of factors influencing it, it is important to adjust organizational practices to individual needs. Older workers who exhibit low levels of functioning, or who are at risk of experiencing it, will welcome HR initiatives that maintain the current levels or accommodate the lost resources (e.g., flexible work arrangement, reduced workload) (Kooij et al., 2014). Age-diversity practices, due to their flexibility to answer to workers’ idiosyncrasy, can promote the engagement of older workers experiencing a decline in work ability.

Finally, the fourth hypothesis proposed the moderated mediation model. Findings show that the effect of age-diversity practices on the preference for early or late retirement via work engagement is stronger for lower levels of work ability. This means that older workers who experience low work ability are more likely to retire later if they are more engaged in their work due to the existence of age-sensitive HR practices in their organization that support and value them. On the contrary, the lack of age-diversity practices may be interpreted by older workers as a sign that the organization does not support them, which in turn will lead them to disengage from the workforce and encourage them to retire earlier than expected.

From a societal perspective, governmental strategies to extend working lives (e.g., increasing statutory retirement age, penalizing early retirement exits) might, on the one hand, provide workers with more opportunities to continue working and, on the other hand, force workers to stay in the labor market, despite their health problems or lack of motivation to work (Flynn, 2010). This means that older workers who feel obligated to work longer will show increases in work disengagement (Damman et al., 2013), challenging organizations’ strategies to encourage workers to work longer. So, age-diversity practices appear to play an important role in providing opportunities for workers to achieve higher levels of functioning and feel motivated to continue working, as well as in supporting individuals to maintain and utilize their existing resources (Zaleska and de Menezes, 2007; Kooij et al., 2014; van Dalen et al., 2015).

Thus, in short, by accommodating age-related changes in workers’ capacities and skills (Ilmarinen, 2001; Truxillo et al., 2015), age-diversity practices can foster older workers’ engagement and their desire to retire later. In that manner, organizations will retain the best talents that are already part of the organization, but will also be able to attract and recruit new talents, achieving an engaged age-diverse workforce.

### Theoretical and Practical Implications

This study makes some valuable academic contributions, namely to the HRM, work engagement, and retirement-related literatures. The results contribute to the HRM literature by showing that practices that are developed taking into account the age-related changes that occur throughout life can benefit older workers’ engagement. These practices are not specific to the age group of older workers, but are instead sensitive to the gains and losses that occur more frequently in this phase. In line with SOC theory (Baltes and Baltes, 1990), age-diversity practices can help individuals to reach higher levels of functioning, to maintain current levels, or to return to earlier levels of functioning, and to function adequately at lower levels (Baltes et al., 1999; Kooij et al., 2014). Thus, age-diversity practices help workers to employ different SOC strategies according to their goals and needs and, therefore, they will become more engaged and committed.

We extend earlier research on work engagement and retirement by showing that workers’ engagement affects retirement intentions. Work engagement is a mechanism that influences a range of positive outcomes, such as organizational commitment, health, and performance (Christian et al., 2011), and can also encourage older workers to postpone retirement. Besides this direct effect, there is a mediating effect of work engagement in the relationship between organizational practices and retirement. This finding shows that this fulfilling, positive state of mind explains how organizations can encourage workers to stay in employment longer.

Finally, the results also contribute to the existing literature on work engagement and retirement by showing the moderating role of work ability in the proposed model. When workers experience low work ability, the relevance of age-sensitive HR practices becomes even more salient. Age-diversity practices are perceived as a signal that the organization still value older workers’ contributions, although there is a gap between personal capacities and work demands. As long as organizations provide working conditions that support older workers, such as age-diversity practices, low work ability will not necessarily result in early retirement (Oksanen and Virtanen, 2012). In fact, despite the perception of low levels of functioning, older workers can still be engaged in their work and want to contribute to organizational success.

Empirical findings reveal important practical implications, especially in a context of an aging workforce. Findings suggest that HR managers can implement age-diversity practices to facilitate work engagement among older workers, especially for those who are most vulnerable due to their low work ability. These results highlight the importance of periodically (e.g., annually) evaluating the engagement and work ability of all workers, including older workers. Many organizations already conduct surveys to evaluate workplace climate or workers’ satisfaction, with the purpose of identifying and implementing strategies for retaining talents and enhancing productivity. Organizations can benefit from measuring these two constructs (i.e., work engagement and work ability) when surveying their workers in order to identify and monitor their needs, and to create practices that meet these needs. Data from these internal surveys can help organizations to determine which groups of workers are at risk of retiring earlier than the statutory retirement age, due either to low work ability or to low work engagement, and adopt the necessary initiatives for a sustainable late career.

For example, in a manufacturing company, older assembly line workers may be exposed to physically demanding tasks, while those in the accounting department may be exposed to prolonged use of visual display terminals. The first group of older workers might benefit from accommodative practices such as flexible work arrangements (e.g., compressed work week, exemption from night shifts). An individual from the second group that has eye strain might benefit from a set of maintenance practices focused on an ergonomic adjustment of work (e.g., make the monitor’s and room brightness match by reducing glare from windows) and training on how to prevent visual discomfort when working at the computer. Such age-diversity practices might encourage workers to use SOC strategies to adapt to changes in work ability, promote engagement, and ensure that late career is a positive experience for individuals.

These bundles of development, maintenance, utilization and accommodative practices can contribute to workers’ engagement, and engaged workers will likely prefer to retire later, which is beneficial for the organization. First, an engaged age-diverse workforce can contribute to better organizational outcomes, such as performance, since workers will feel valued and committed with their organization (Boehm et al., 2014). Second, organizations can capture important knowledge that older workers possess that might be advantageous or detrimental for organizational success, and also ensure that this knowledge is transferred to the workers who will succeed those who are retiring in the near future (e.g., workforce planning, succession planning, mentoring). Finally, age-diversity practices can make organizations more appealing for candidates of all ages, which will facilitate the attraction and recruitment of individuals that meet organizational requirements. This will result in an age-diverse workplace with a range of different skills, experience, and perspectives, which might contribute to greater creativity and innovation in teams (e.g., Wegge et al., 2012). Hence, age-diversity practices can help organizations to extend working lives, thus responding proactively to the challenges of an aging workforce.

### Limitations and Future Research

This study highlighted the relevance of age-diversity practices, work ability, and work engagement in retirement preferences, expanding current knowledge about the organizational role in the retirement decision-making process. However, it also has some limitations. First, data were collected at one point in time and, therefore, the conclusions need to be interpreted with caution since in cross-sectional studies it is not possible to identify the causal order of the relationships under study. Future studies with longitudinal designs are welcomed to explore causal effects between these constructs and also to follow workers’ perceptions about age-diversity practices over time. A second limitation pertains the non-representative sample of this study for older Portuguese workers, which limits the generalizability of the findings. Data were collected with a convenience sample focusing on heterogeneity, with the purpose of increasing the variety of jobs, individual (e.g., age, gender, education) and organizational (e.g., HR practices, dimension, industry) characteristics. In the future, it would be important to replicate the moderated mediation model in a representative sample of older Portuguese workers. A third limitation of this study is that all participants were from one country (Portugal). Since there are retirement-related particularities in each country, future research should consider other cultures for more generalized results.

Also, we measured perceived rather than actual practices as we were interested in understanding how workers perceive the bundles of HR practices implemented by their organization. However, actual practices can vary across organizations according to their business strategies and the environmental constraints and contingencies. For instance, some organizations may implement essentially development practices for older workers, providing training and internal promotion opportunities, while other organizations may offer mainly accommodative practices, such as workload reductions. Thus, it would be important to apply the case study design to explore how actual and perceived age-supportive HR practices influence work engagement and retirement preferences.

In this study, we conceptualized the preference for early or late retirement in terms of individual preferences and expectations about retirement timing and, therefore, operationalized it using a subjective measure. Future research on this topic could operationalize early, on-time, and late retirement using objective criteria such as age, years of service, and eligibility (e.g., the age at which one becomes eligible for a government pension), and examine the influence of age-diversity practices, work engagement, and work ability in these measures.

Finally, it would also be interesting to examine differences across industries or occupations. Different industries are represented in this study, but the sample size does not allow comparisons among them. In the future, the proposed model can be examined comparing, for instance, the health sector with manufacturing, providing some extra insight about the importance and influence of age-diversity practices and work engagement in these specific contexts.

## Data Availability

The datasets generated for this study are available on request to the corresponding author.

## Ethics Statement

This study was carried out in accordance with the guidelines of the Code of Ethical Conduct on Research of the Ethics Committee of the ISCTE – University Institute of Lisbon and of the Code of Ethics of the Order of Portuguese Psychologists. All participants gave written informed consent in accordance with the Declaration of Helsinki. Respondents were provided with information about the purpose of the study and were assured of anonymity and confidentiality. Participants were also informed that their participation was voluntary and of their right to withdraw participation at any time without prejudice from any party. The protocol was approved retrospectively by the Ethics Committee of the ISCTE – University Institute of Lisbon.

## Author Contributions

All authors listed have made a substantial, direct and intellectual contribution to the work, and approved it for publication.

## Conflict of Interest Statement

The authors declare that the research was conducted in the absence of any commercial or financial relationships that could be construed as a potential conflict of interest.
